# Adenovirus in a Kidney Transplant Recipient

**DOI:** 10.1016/j.xkme.2023.100605

**Published:** 2023-01-25

**Authors:** Erik L. Lum, Jonathan Zuckerman, Pryce Gaynor, Suphamai Bunnapradist

**Affiliations:** a Division of Nephrology, Department of Medicine, UCLA David Geffen School of Medicine, Los Angeles, CA; b Department of Pathology, UCLA David Geffen School of Medicine, Los Angeles, CA; c Division of Infectious Disease, Department of Medicine, ULCA David Geffen School of Medicine, Los Angeles, CA

A kidney transplant recipient in his 30s with kidney failure secondary to IgA nephropathy presented to the clinic with gross hematuria. He had received a deceased donor kidney transplant 16 months earlier from a donor in their 20s. He received basiliximab for induction and experienced immediate graft function. He achieved a baseline creatinine level of 1.3 mg/dL and was maintained on prednisone, tacrolimus, and mycophenolate mofetil. He developed transient BK DNAemia 3 months after transplant, with a peak copy level of 2,300 copies that responded to reduction in mycophenolate mofetil.

**Figure 1:**
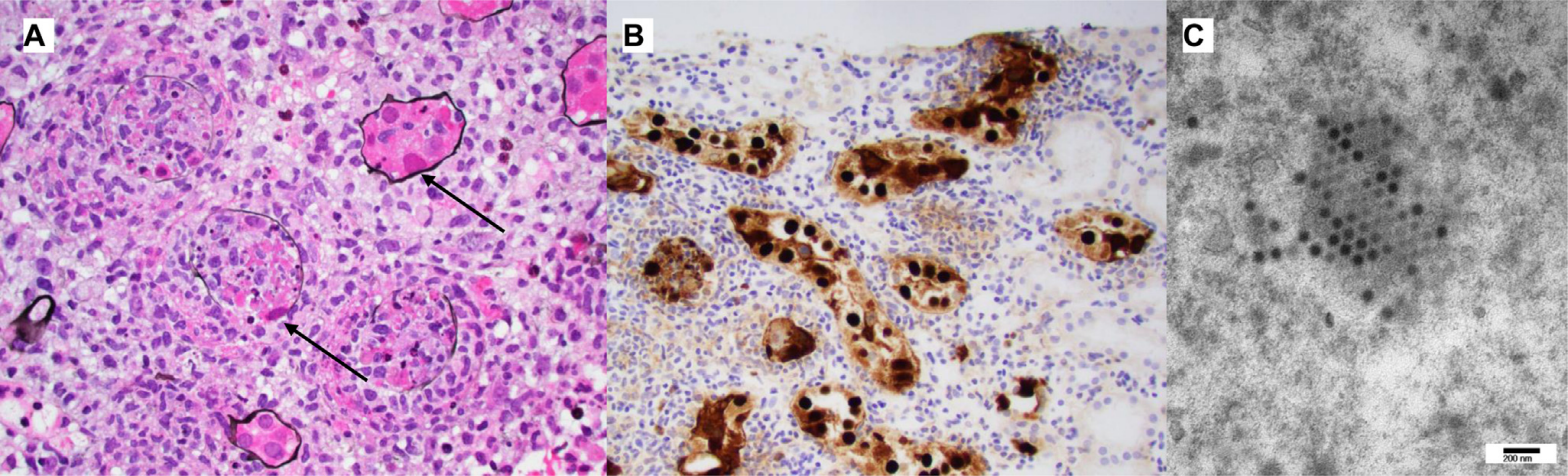
Kidney biopsy findings. (A) Diffuse tubulointerstitial inflammation with a vaguely granulomatous appearance, associated tubular rupture, and nuclear viral inclusions (arrows) (400x; Jones Silver Stain). (B) Adenovirus immunohistochemical stain (200x). (C) Electron micrograph demonstrating adenovirus virions (59,500x).

Three weeks before presentation, he developed low-grade fevers and poor appetite, followed by a dry cough 1 week later and subsequent gross hematuria. Testing result for severe acute respiratory syndrome coronavirus 2 was negative. On physical examination, his temperature was 38.1 °C, with a blood pressure of 111/71 mm Hg and a pulse rate of 107 bpm. Examination was otherwise unremarkable. Laboratory testing was notable for an increase in serum creatinine level to 2.25 mg/dL; 2+ proteinuria and hematuria on dipstick, with a urine protein-to-creatinine ratio of 0.7; and adenovirus polymerase chain reaction quantification of 977,000 copies/mL. A kidney transplant ultrasound was unremarkable. Kidney biopsy showed adenovirus nephritis (Fig 1).^1^ Mycophenolate mofetil was stopped, and he received cidofovir 4 mg/kg on days 0, 2, and 9 with probenecid premedication.^2^ His adenovirus polymerase chain reaction quantification declined to 0 copies/mL with treatment, and his creatinine level improved to baseline.
